# Efficacy and safety of larotrectinib in patients with TRK fusion-positive thyroid carcinoma

**DOI:** 10.1530/EJE-21-1259

**Published:** 2022-03-25

**Authors:** Steven G Waguespack, Alexander Drilon, Jessica J Lin, Marcia S Brose, Ray McDermott, Mohammed Almubarak, Jessica Bauman, Michela Casanova, Anuradha Krishnamurthy, Shivaani Kummar, Serge Leyvraz, Do-Youn Oh, Keunchil Park, Davendra Sohal, Eric Sherman, Ricarda Norenberg, Josh D Silvertown, Nicoletta Brega, David S Hong, Maria E Cabanillas

**Affiliations:** 1 The University of Texas MD Anderson Cancer Center, Houston, Texas, USA; 2 Memorial Sloan Kettering Cancer Center, New York, New York, USA; 3 Weill Cornell Medical College, New York, New York, USA; 4 Massachusetts General Hospital, Boston, Massachusetts, USA; 5 Harvard Medical School, Boston, Massachusetts, USA; 6 Sidney Kimmel Cancer Center of Jefferson University Health, Philadelphia, Pennsylvania, USA; 7 St Vincent’s University Hospital and Cancer Trials Ireland, Dublin, Ireland; 8 West Virginia University, Morgantown, West Virginia, USA; 9 Fox Chase Cancer Center, Philadelphia, Pennsylvania, USA; 10 Paediatric Oncology Unit, Fondazione IRCCS Istituto Nazionale dei Tumori, Milan, Italy; 11 UPMC Hillman Cancer Center, University of Pittsburgh, Pittsburgh, Pennsylvania, USA; 12 Stanford Cancer Center, Stanford University, Palo Alto, California, USA; 13 Charité – Universitätsmedizin Berlin, Berlin, Germany; 14 Seoul National University Hospital, Cancer Research Institute, Seoul National University College of Medicine, Integrated Major in Innovative Medical Science, Seoul National University Graduate School, Seoul, South Korea; 15 Samsung Medical Center, Sungkyunkwan University School of Medicine, Seoul, South Korea; 16 University of Cincinnati, Cincinnati, Ohio, USA; 17 Chrestos Concept GmbH & Co. KG, Essen, Germany; 18 Bayer HealthCare Pharmaceuticals, Inc., Toronto, Canada; 19 Bayer S.p.A, Milan, Italy

## Abstract

**Objective:**

Larotrectinib is a highly selective tropomyosin receptor kinase (TRK) inhibitor with demonstrated efficacy across various TRK fusion-positive solid tumours. We assessed the efficacy and safety of larotrectinib in patients with TRK fusion-positive thyroid carcinoma (TC).

**Methods:**

We pooled data from three phase I/II larotrectinib clinical trials (NCT02576431, NCT02122913, and NCT02637687). The primary endpoint was the investigator-assessed objective response rate (ORR) per Response Evaluation Criteria in Solid Tumors v1.1. Secondary endpoints included duration of response (DoR), progression-free survival (PFS), overall survival (OS), and safety. Data cut-off: July 2020.

**Results:**

Twenty-nine patients (median age: 60; range: 6–80) with TRK fusion-positive TC were treated. Tumour histology was papillary (PTC) in 20 (69%) patients, follicular (FTC) in 2 (7%), and anaplastic (ATC) in 7 (24%) patients. Among 28 evaluable patients, ORR was 71% (95% CI: 51–87); best responses were complete response in 2 (7%) patients, partial response in 18 (64%), stable disease in 4 (14%), progressive disease in 3 (11%), and undetermined in 1 (4%) due to clinical progression prior to the first post-baseline assessment. ORR was 86% (95% CI: 64–97) for PTC/FTC and 29% (95% CI 4–71) for ATC. Median time to response was 1.87 months (range 1.64–3.68). The 24-month DoR, PFS, and OS rates were 81, 69, and 76%, respectively. Treatment-related adverse events were mainly grades 1–2.

**Conclusion:**

In TRK fusion-positive TC, larotrectinib demonstrates rapid and durable disease control and a favourable safety profile in patients with advanced disease requiring systemic therapy.

**Significance statement:**

NTRK gene fusions are known oncogenic drivers and have been identified in various histologies of thyroid carcinoma, most commonly in papillary thyroid carcinoma. This is the first publication specifically studying a TRK inhibitor in a cohort of TRK fusion-positive thyroid carcinoma patients. In the current study, the highly selective TRK inhibitor larotrectinib showed durable antitumour efficacy and a favourable safety profile in patients with TRK fusion-positive thyroid carcinoma. Our findings show that patients with advanced non-medullary thyroid carcinoma who may require systemic therapy could be considered for testing for gene fusions by next-generation sequencing.

## Introduction

Tropomyosin receptor kinase (TRK) proteins are a family of receptors that are vital for normal nervous system functioning (1). The three structurally related TRK receptors, TRKA, TRKB, and TRKC, are encoded by three distinct genes: *NTRK1*, *NTRK2*, and *NTRK3*, respectively. Recurrent *NTRK* gene fusion events have been reported in a diverse range of adult and paediatric cancers (2, 3, 4, 5, 6). These events have been detected at frequencies ranging from less than 1 to 25%, depending on the cancer type, and at more than 90% in some rare tumours (7). Indeed, papillary thyroid carcinoma (PTC) was one of the first tumour types in which *NTRK1* fusions were identified (7, 8). *NTRK* gene fusions tend to be the primary oncogenic drivers in tumours that harbour them and co-occurrence with other known oncogenic alterations, including *BRAF* mutations, is uncommon (9).

TRK fusion-positive thyroid carcinoma (TC) is more commonly associated with a younger age of diagnosis but can be identified across the age spectrum (10, 11, 12). Depending on the population, approximately 5–25% of PTC cases in paediatric patients are reported to harbour* NTRK* gene fusions (10, 12, 13, 14, 15) while about 6% of adult PTCs have *NTRK* gene fusions (12). TRK fusion-positive TC appears to have unique morphologic characteristics, such as a follicular growth pattern, and it may be more associated with locoregional and distantly metastatic disease (10, 11, 12, 16, 17). In a recent genomic analysis of 126 patients with anaplastic thyroid carcinoma (ATC), three patients (2.4%) with ATC were found to harbour an *NTRK* gene fusion (18). In other recent studies, no *NTRK* gene fusions were identified in ATC or follicular thyroid carcinoma (FTC), although the sample size was small (11, 12).

Larotrectinib is a first-in-class, CNS-active, highly selective TRK inhibitor that is approved in more than 40 countries for adult and paediatric patients with TRK fusion-positive cancer (19, 20). Larotrectinib has demonstrated durable antitumour efficacy in a pooled analysis of 55 adults and/or children with various cancers from three phase I/II trials (21). This efficacy was sustained after further follow-up, and in an expanded patient population (*n* = 159), the objective response rate (ORR) was 79% (95% CI: 72–85) and the median duration of response was 35.2 months (95% CI: 22.8–not estimable) (22). Larotrectinib was well tolerated, with most adverse events (AEs) being grade 1 or 2 (22). The proportion of patients who experienced dose reductions or discontinued treatment due to treatment-related AEs was 8 and 2%, respectively (22). In the current study, we evaluate the efficacy and safety of larotrectinib in the subset of patients with TRK fusion-positive TC.

## Subjects and methods

### Study design

For this analysis, we included patients with measurable, locally advanced, or metastatic TC harbouring an *NTRK* gene fusion who were treated with larotrectinib in one of three clinical trials: a phase II ‘basket’ trial (NAVIGATE) in adults and adolescents (aged ≥12 years) with advanced solid TRK fusion-positive tumours (NCT02576431), a phase I trial in adults aged ≥18 years with advanced solid tumours (NCT02122913), and a phase I/II trial (SCOUT) in paediatric patients (aged <21 years) with advanced solid or primary CNS tumours (NCT02637687). The full methodology for these studies has been previously described (21, 22, 23); study eligibility criteria are summarised in Appendix 1 of the Data Supplement (see section on supplementary materials given at the end of this article).

In brief, larotrectinib was administered at 100 mg twice daily in adults (or 100 mg/m^2^ twice daily in paediatric patients) and continued until disease progression, withdrawal of the patient from the study, or unacceptable toxicity. Patients could continue treatment beyond progression if they were still benefitting in the judgement of the investigator. Tumour histology was locally assessed and* NTRK* gene fusions were detected by local molecular testing in Clinical Laboratory Improvement Amendments-certified or similarly accredited laboratories. All study protocols were approved by the site-specific institutional review board or independent ethics committees and were compliant with the International Ethical Guidelines for Biomedical Research Involving Human Subjects, Good Clinical Practice guidelines, the Declaration of Helsinki, and local laws. Written informed consent was obtained prior to study entry.

### Study endpoints

The primary endpoint was ORR assessed by the investigator according to Response Evaluation Criteria in Solid Tumors (RECIST) version 1.1 (24). Secondary endpoints included duration of response (DoR), progression-free survival (PFS), overall survival (OS), and safety outcomes.

### Study assessments

Tumours were assessed using a combination of CT, MRI, and clinical measurement at baseline, every 8 weeks for 12 months, then every 12 weeks thereafter until disease progression. For paediatric patients in the SCOUT trial, in the absence of disease progression, tumour assessment was decreased to every 6 months after 2 years of treatment.

### Statistical analysis

DoR, PFS, and OS were estimated using Kaplan–Meier analysis. Confidence intervals (95% CIs) were calculated using the Clopper–Pearson method.

## Results

### Patient population

A total of 29 patients with TRK fusion-positive TC were identified at the data cut-off of 20 July 2020. Tumour histology, based on local assessment, was PTC in 20 (69%) patients and FTC in 2 (7%), collectively referred to as differentiated TC (DTC), and seven patients (24%) were classified as ATC (undifferentiated carcinoma). Among the ATC patients, two patients were transformed cases from PTC. Two patients classified as ATC by the local investigators had poorly differentiated TC (PDTC), as did one patient classified as PTC (Supplementary Table 1). Patient demographics and clinical characteristics are summarised in Table 1.

**Table 1:** Demographics and clinical characteristics of 29 patients with advanced TRK fusion-positive thyroid carcinoma treated with larotrectinib.

Characteristics	All	PTC/FTC	ATC
*n*	29	22	7
Age at study enrolment, median (range), years	60.0 (6.0–80.0)	58.0 (6.0–80.0)	64.0 (49.0–77.0)
Paediatric (<18 years)	2 (7)	2 (9)	0
Adult (≥18 years)	27 (93)	20 (91)	7 (100)
Sex, n (%)			
Female	20 (69)	15 (68)	5 (71)
Male	9 (31)	7 (32)	2 (29)
ECOG performance status, *n* (%)			
0	12 (41)	11 (50)	1 (14)
1	12 (41)	8 (36)	4 (57)
2	4 (14)	3 (14)	1 (14)
3	1 (3)	0	1 (14)
Cancer subtype, *n* (%)			
Papillary*	20 (69)	20 (91)	0
Follicular	2 (7)	2 (9)	0
Anaplastic*	7 (24)	0	7 (100)
Brain metastases at baseline, *n* (%)			
Yes	4 (14)	4 (18)	0
No	25 (86)	-	-
Prior therapies^†^, *n* (%)			
Surgery	29 (100)	22 (100)	7 (100)
Radiotherapy	17 (59)	12 (55)	5 (71)
RAI	23 (79)	21 (95)	2 (29)
Systemic therapy^†‡^	16 (55)	13 (59)	3 (43)
Tyrosine kinase inhibitors	11 (38)	11 (50)	0
Immunotherapy	2 (7)	2 (9)	0
Chemotherapy	3 (10)	0	3 (43)
Number of prior systemic therapies, *n* (%)			
0	13 (45)	9 (41)	4 (57)
1	7 (24)	4 (18)	3 (43)
2	7 (24)	7 (32)	0
≥3	2 (7)	2 (9)	0
*NTRK* gene, *n* (%)			
*NTRK1*	13 (45)	10 (45)	3 (43)
*NTRK3*	16 (55)	12 (55)	4 (57)

*One patient classified as PTC and two patients classified as ATC had PDTC. ^†^Patients may be counted in more than 1 category. ^‡^Including cabozantinib, cisplatin, doxorubicin, ipilimumab, lenvatinib, paclitaxel, pazopanib, pembrolizumab, sorafenib, sunitinib and trametinib.

ATC, anaplastic thyroid carcinoma; ECOG, Eastern Cooperative Oncology Group; FTC, follicular thyroid carcinoma; *NTRK*, neurotrophic tyrosine receptor kinase; PDTC, poorly differentiated thyroid carcinoma; PTC, papillary thyroid carcinoma; RAI, radioactive iodine; TRK, tropomyosin receptor kinase.

The median age at study enrolment was 60 years (range: 6–80 years) and 2 (7%) patients were children (6 and 13 years old). A total of four (14%; three PTC and one FTC) patients had CNS metastases at baseline, three (75%) of whom had received prior radiation to the brain. Overall, *NTRK1*and* NTRK3* gene fusions were identified in 45 and 55% of patients, respectively; there were no *NTRK2* gene fusions. *NTRK*gene fusions were identified by RNA-based next-generation sequencing (NGS) in 17 (59%) patients (*NTRK1*: *n*  = 8; *NTRK3*: *n*  = 9), DNA NGS in 5 (17%) patients (*NTRK1*: *n*  = 2; *NTRK3*: *n*  = 3), and DNA and RNA NGS in 7 (24%) patients (*NTRK1*: *n*  = 3; *NTRK3*: *n*  = 4). The fusion partners for the 13 patients with *NTRK1* gene fusions were *TPM3* (*n* = 4), *TPR* (*n* = 4), *IRF2BP2* (*n* = 2), *NFASC* (*n* = 1), *PPL* (*n* = 1), and *DIAPH1* (*n* = 1), and for the 16 patients with *NTRK3* gene fusions, they were *ETV6* (*n* = 14) and *EML4* (*n* = 2) (Supplementary Table 1).

As per study eligibility criteria, all enrolled patients had either received prior standard therapy, had a tumour for which there is no standard therapy, or in the opinion of the investigator were considered unlikely to derive clinically meaningful benefit from standard therapy. All patients who had received prior standard therapy had either progressed or were unresponsive to treatment. Prior therapies included surgery in all 29 (100%) patients, radiotherapy in 17 (59%) patients, radioactive iodine (RAI) in 23 (79%) patients, and systemic therapy in 16 (55%) patients; 9 (31%) patients had received ≥2 prior systemic therapies. Two patients (7%) had received prior immunotherapy, both with progressive disease as the best response.

### Efficacy

**Figure 1:**
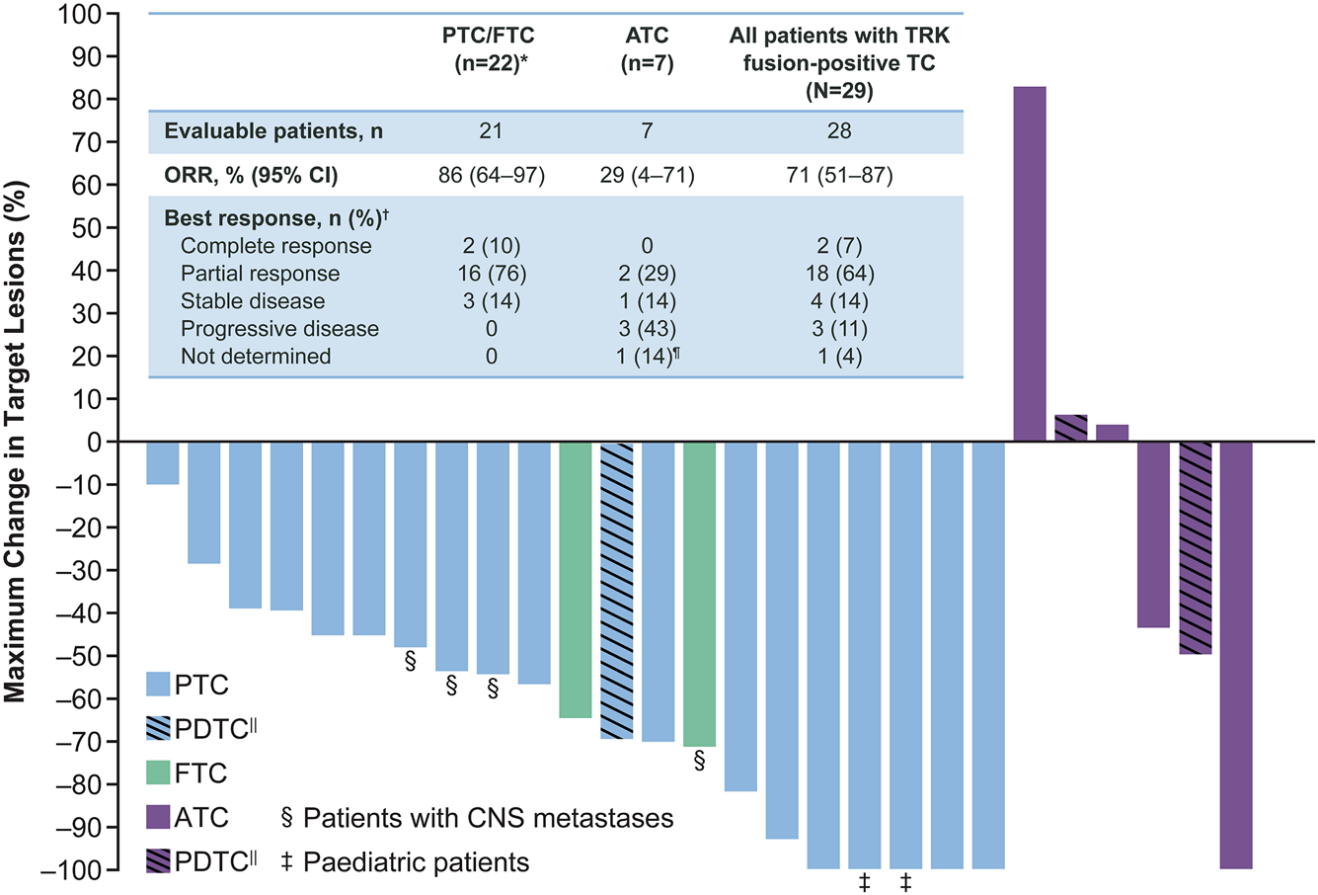
Response to larotrectinib. A waterfall plot of the maximum change in target lesions following treatment with larotrectinib in patients with advanced TRK fusion-positive thyroid carcinoma. The table depicts the overall response in both target and non-target lesions, and the waterfall plot depicts the maximum change in target lesions. *One patient with papillary TC was not evaluable for assessment of tumour response. ^†^Investigator assessment based on RECIST version 1.1. ^||^Three PDTCs, two in the anaplastic group and one in the papillary group. ^¶^One patient with anaplastic TC was evaluable, but the response could not be determined because they had clinical disease progression prior to the first tumour response assessment. ATC, anaplastic thyroid carcinoma; FTC, follicular thyroid carcinoma; ORR, objective response rate; PDTC, poorly differentiated thyroid carcinoma; PTC, papillary thyroid carcinoma; TC, thyroid carcinoma; TRK, tropomyosin receptor kinase. A full colour version of this figure is available at https://doi.org/10.1530/EJE-21-1259.

**Figure 2:**
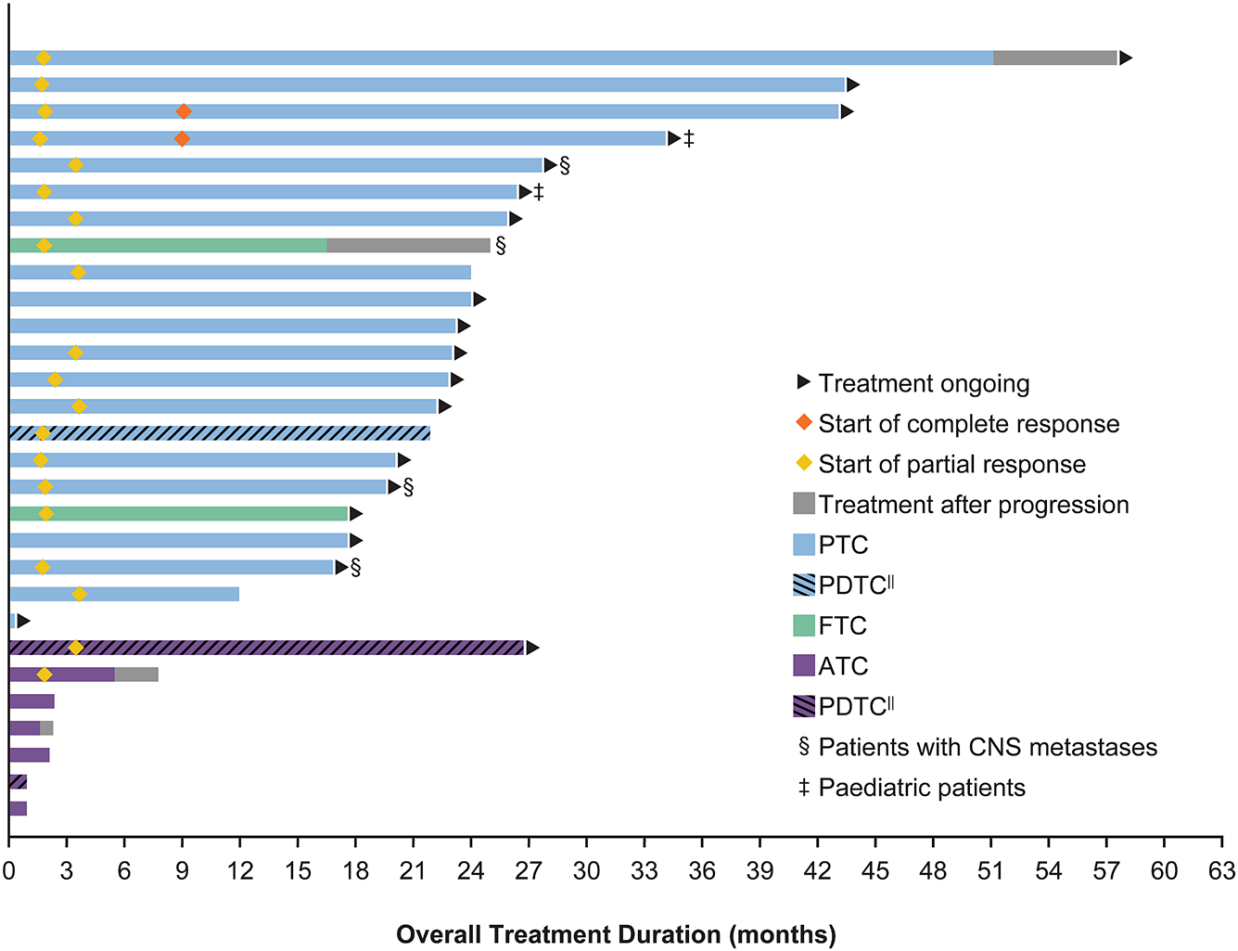
Treatment duration. A swimmer plot of the treatment duration in patients with advanced TRK fusion-positive thyroid carcinoma treated with larotrectinib. ^||^Three PDTCs, two in the anaplastic group and one in the papillary group. ATC, anaplastic thyroid carcinoma; FTC, follicular thyroid carcinoma; PDTC, poorly differentiated thyroid carcinoma; PTC, papillary thyroid carcinoma; TRK, tropomyosin receptor kinase. A full colour version of this figure is available at https://doi.org/10.1530/EJE-21-1259.

Among 28 evaluable patients, the ORR in both target and non-target lesions was 71% (95% CI: 51–87) (Fig. 1); 2 (7%) patients had a complete response (CR; including 1 paediatric patient), 18 (64%) had a partial response (PR), 4 (14%) had stable disease (SD), and 3 (11%) had progressive disease (PD). All three patients with progressive disease had ATC, including one PDTC patient. In 1 (4%) patient with ATC, response could not be determined due to clinical disease progression prior to the first post-baseline assessment. One (3%) of the patients with PTC was considered not evaluable for the assessment of tumour response as they had not been on treatment long enough for the first evaluation by the data cut-off. Among patients with an objective response (*n* = 20), the median time to response (RECIST v1.1) was 1.87 months (range: 1.64–3.68) (Fig. 2), which was the time of the first post-baseline assessment in most cases.

Among the 22 patients with DTC (PTC and FTC), the ORR was 86% (95% CI: 64–97). Among 19 evaluable patients with PTC, 2 had a CR (including 1 paediatric patient), 14 had a PR (including 1 PDTC patient), and 3 had SD. Both patients with FTC had a PR. Among the seven patients with ATC, the ORR in both target and non-target lesions was 29% (95% CI: 4–71); two had a PR (including one PDTC patient), one had SD, three had PD (including one PDTC patient), and in one patient the response could not be determined (Fig. 1).

Among the 13 patients with DTC who had one or more prior lines of systemic therapy, the ORR was 92%. The disease control rate (DCR) at 24 weeks for DTC (PTC and FTC) was 91% (95% CI: 71–99). The DCR at 24 weeks for ATC was 29% (95% CI: 4–71). All four patients with CNS metastases at baseline had a PR as the best overall response. Two patients had measurable intracranial disease, with intracranial tumour reductions of 14 and 46%; both had received radiotherapy ~14–15 months prior to starting larotrectinib. Of the four patients with baseline CNS metastases, one had progressed after 17 months while three had not progressed at the data cut-off, with PFS censored at 17, 20, and 27 months, respectively.

Duration of treatment ranged from 0.26+ to 57.5+ months (Fig. 2). By the data cut-off, treatment was ongoing in 19 (66%; 17 PTC, 1 FTC, and 1 ATC) patients. Six patients had progressed on treatment, of which four patients continued treatment post-progression for ≥20 days due to perceived clinical benefit. Two patients died on therapy (one with PTC/PDTC and one with ATC). Two patients discontinued treatment due to their own decision and were alive without progression at the data cut-off. One patient discontinued therapy at the recommendation of their physician and was alive at data cut-off without post-study disease assessments.

**Figure 3:**
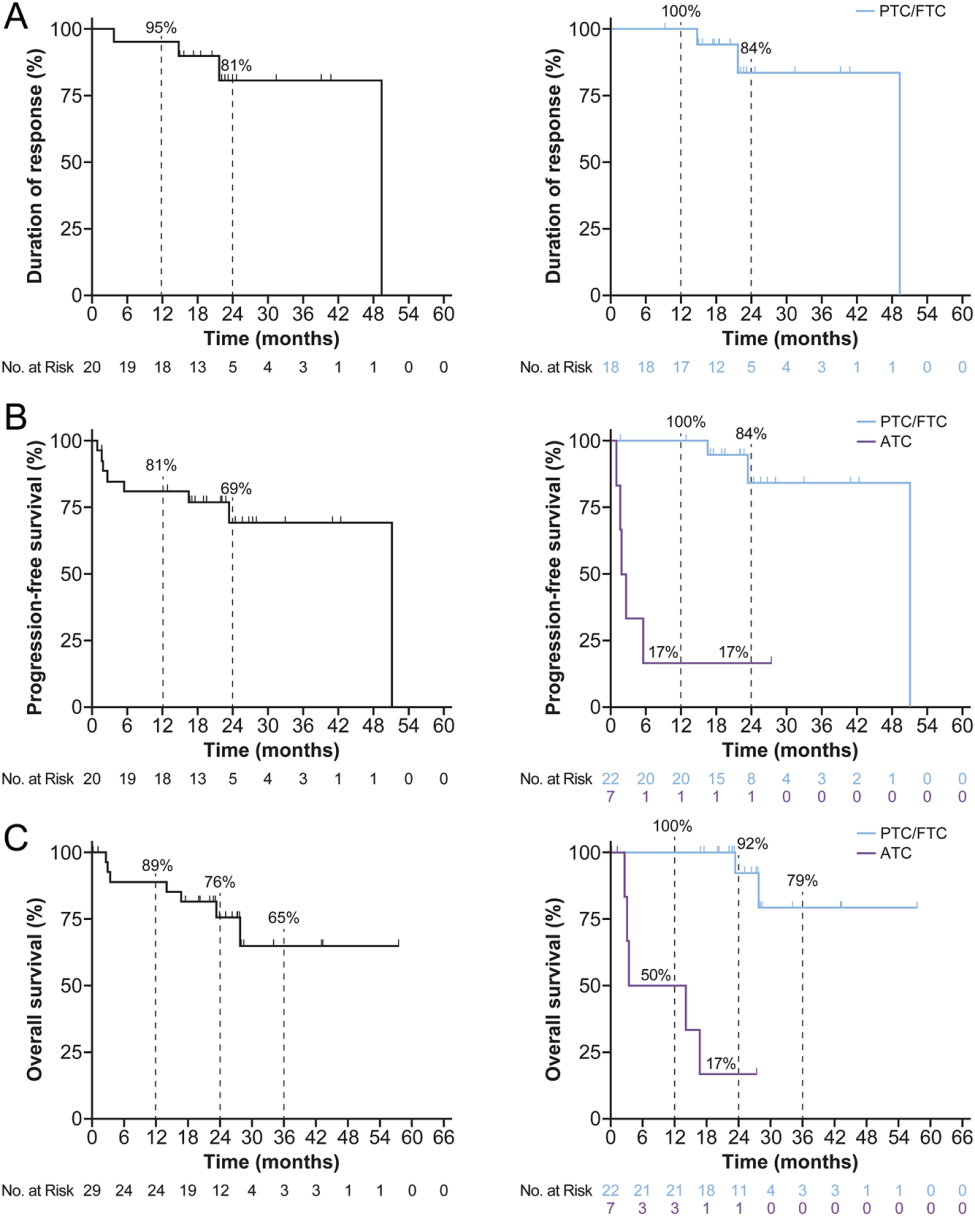
Duration of response and survival. Kaplan–Meier curves of (A) duration of response^†^, (B) progression-free survival^‡^, and (C) overall survival^‡^ in patients with advanced TRK fusion-positive thyroid carcinoma treated with larotrectinib. The left panels show data for the entire study group and the right panels show the data based on histology. ^†^Duration of response Kaplan-Meier curve only includes the patients who experienced a response. ATC duration of response Kaplan-Meier curve is not shown due to too few patients. ^‡^The one patient in the ATC group with a durable response had PDTC. ATC, anaplastic thyroid carcinoma; FTC, follicular thyroid carcinoma; PDTC, poorly differentiated thyroid carcinoma; PTC, papillary thyroid carcinoma; TRK, tropomyosin receptor kinase. A full colour version of this figure is available at https://doi.org/10.1530/EJE-21-1259.

The Kaplan–Meier estimated DoR rate for all responders (*n* = 20) at 12 and 24 months was 95 and 81%, respectively (Fig. 3A). The PFS rate at 12 and 24 months was 81 and 69%, respectively (Fig. 3B). The proportion of patients alive after 12, 24, and 36 months was 89, 76, and 65%, respectively (Fig. 3C). Among the patients with PTC/FTC, the DoR rate at 12 and 24 months was 100 and 84%, respectively. The PFS rate at 12 and 24 months was 100 and 84%, respectively. The OS rate at 12, 24, and 36 months was 100, 92, and 79%, respectively.

**Figure 4:**
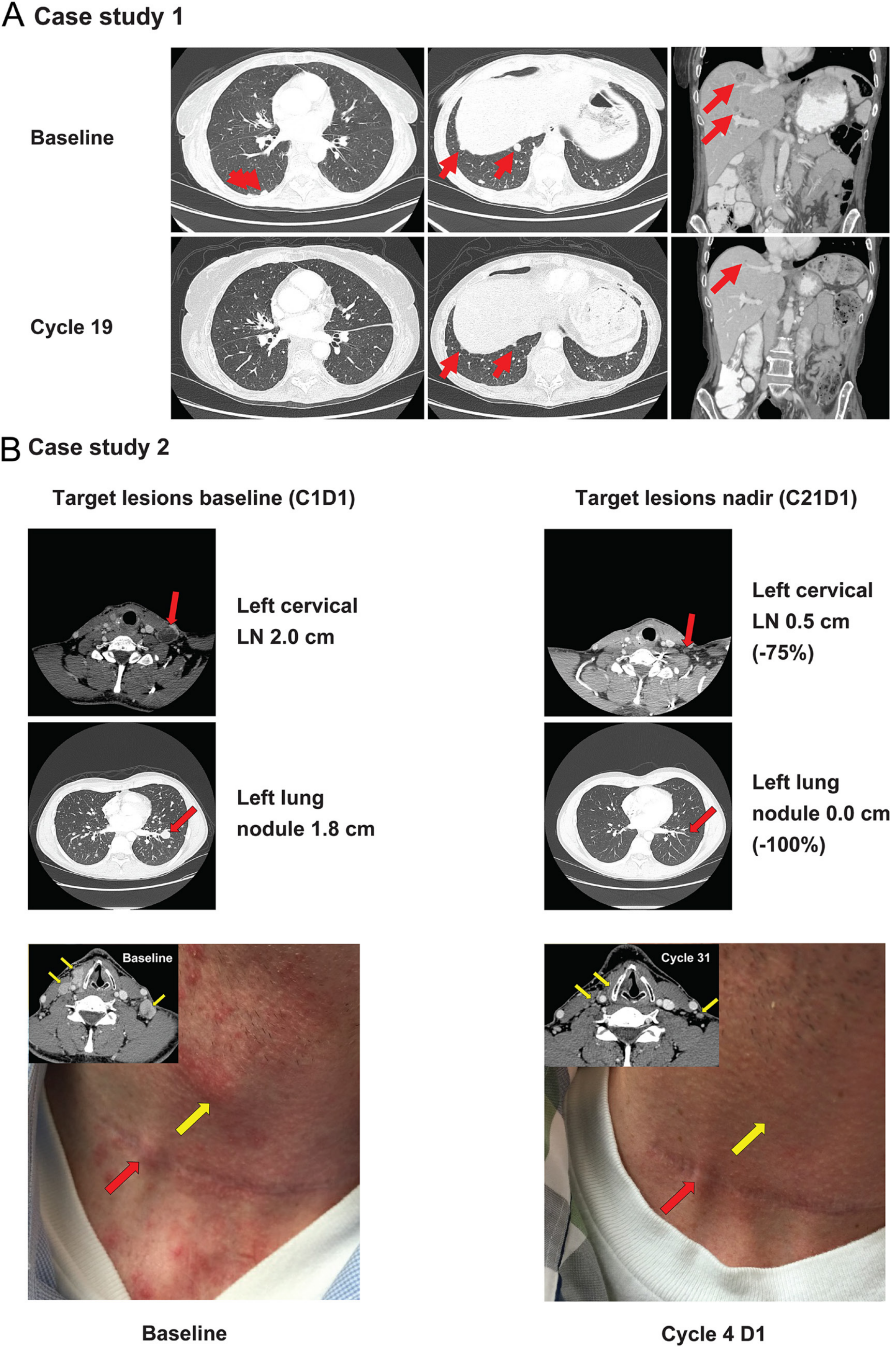
Case studies of patients with advanced TRK fusion-positive thyroid carcinoma treated with larotrectinib. Contrast-enhanced CT images demonstrating response in target and non-target lesions. (A) An adult female patient was diagnosed with PDTC with an *ETV6-NTRK3* gene fusion. The red arrows indicate lung and liver metastases that responded to therapy. (B) An adult male patient was diagnosed with PTC with an *ETV6-NTRK3* gene fusion. The red arrows indicate target lesions, and the yellow arrows identify non-target lesions. (C) A paediatric male patient was diagnosed with PTC with an *IRF2BP2-NTRK1* gene fusion and a complete response in a lung target lesion (yellow arrows). LN, lymph node; *NTRK*, neurotrophic tyrosine receptor kinase; PDTC, poorly differentiated thyroid carcinoma; PTC, papillary thyroid carcinoma; TRK, tropomyosin receptor kinase. A full colour version of this figure is available at https://doi.org/10.1530/EJE-21-1259.

**Figure:**
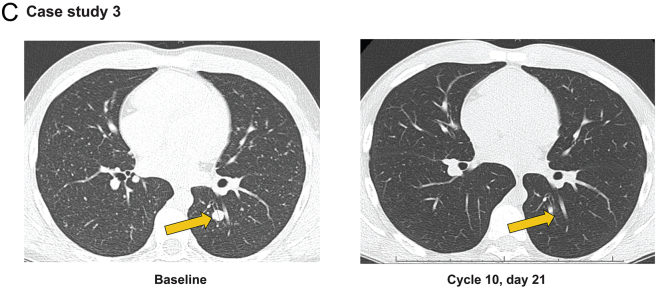

For patients classified as ATC, the DoR rate at 12 months was 50%. Median PFS was 2.2 months (95% CI: 0.9–NE) after a median follow-up of 27.4 months. The 12- and 24-month PFS rates were both 17%. Median OS was 8.8 months (95% CI: 2.6–NE) over a median follow-up of 27.4 months. The OS rate at 12 and 24 months was 50 and 17%, respectively. However, these data are skewed by one patient with PDTC who had a prolonged response to therapy.

### Safety

Treatment-related AEs were reported by 26 (90%) patients, the most common being myalgia, fatigue, dizziness, and elevated liver transaminases. AEs that occurred in ≥15% of patients are shown in Table 2. Treatment-related AEs were mostly grade 1 or 2 and were consistent with the pooled analysis (22). Two (7%) patients experienced grade 3 AEs considered related to larotrectinib (anaemia and decreased lymphocyte count). There were two grade 5 treatment-emergent AEs (7%) that were not considered to be related to larotrectinib; one patient died from a tracheal haemorrhage and one patient died due to progressive thyroid carcinoma. A total of two patients (7%) experienced an AE leading to dose reduction, with dose reductions lasting approximately 2 and 3 weeks, respectively. No patients experienced an AE that resulted in permanent discontinuation of larotrectinib.

**Table 2:** Adverse events occurring in ≥15% of patients with advanced TRK fusion-positive thyroid carcinoma treated with larotrectinib.

Preferred term	Treatment-emergent AEs, *n* (%)				Treatment-related AEs, *n* (%)		
	Grade 1 or 2	Grade 3	Grade 4	Any grade	Grade 3	Grade 4	Any grade
Myalgia	12 (41)	0	0	12 (41)	0	0	8 (28)
Fatigue	10 (34)	0	0	10 (34)	0	0	8 (28)
Nausea	10 (34)	0	0	10 (34)	0	0	3 (10)
Constipation	9 (31)	0	0	9 (31)	0	0	5 (17)
Cough	8 (28)	1 (3)	0	9 (31)	–	–	–
Dizziness	8 (28)	1 (3)	0	9 (31)	0	0	8 (28)
Peripheral oedema	9 (31)	0	0	9 (31)	0	0	4 (14)
ALT increased	8 (28)	0	0	8 (28)	0	0	8 (28)
Anaemia	4 (14)	4 (14)	0	8 (28)	1 (3)	0	2 (7)
AST increased	8 (28)	0	0	8 (28)	0	0	8 (28)
Arthralgia	7 (24)	0	0	7 (24)	0	0	3 (10)
Diarrhoea	4 (14)	3 (10)	0	7 (24)	0	0	3 (10)
Dyspnoea	6 (21)	1 (3)	0	7 (24)	0	0	1 (3)
Leukocyte count decreased	6 (21)	1 (3)	0	7 (24)	0	0	6 (21)
Lymphocyte count decreased	3 (10)	3 (10)	1 (3)	7 (24)	1 (3)	0	2 (7)
Vomiting	7 (24)	0	0	7 (24)	0	0	2 (7)
Headache	6 (21)	0	0	6 (21)	0	0	1 (3)
Pyrexia	6 (21)	0	0	6 (21)	0	0	1 (3)
Hypoaesthesia	5 (17)	0	0	5 (17)	0	0	3 (10)
Hypocalcaemia	2 (7)	2 (7)	1 (3)	5 (17)	0	0	1 (3)
Nasal congestion	5 (17)	0	0	5 (17)	0	0	1 (3)
Pain in extremity	5 (17)	0	0	5 (17)	0	0	2 (7)
Rash	5 (17)	0	0	5 (17)	0	0	3 (10)

Dashes indicate AEs that were not reported to be treatment-related in any patients.

AE, adverse event; ALT, alanine aminotransferase; AST, aspartate aminotransferase; TRK, tropomyosin receptor kinase.

### Case study 1

A 64-year-old woman with a history of chronic obstructive pulmonary disease (COPD) was diagnosed with PDTC derived from PTC and treated with a total thyroidectomy and RAI. One year later, she developed locoregional recurrence and bone metastases. She underwent a left-neck dissection and partial oesophagectomy, followed by external beam radiation to the neck and stereotactic radiation to multiple levels of the vertebral spine. The patient continued to progress and was enrolled in a clinical trial of radiation and immunotherapy but without response. An *ETV6*-*NTRK3* gene fusion was detected through NGS with Oncomine Focus assay, and she began larotrectinib 100 mg twice daily. The patient achieved a PR by cycle 3, with 70% tumour reduction by cycle 19 (Fig. 4A). After 23 completed cycles, larotrectinib was suspended due to COPD complications and pneumonia, which were unrelated to larotrectinib. The patient’s disease progressed, and she died approximately 3 months later.

### Case study 2

A 33-year-old male, diagnosed at 27 years of age, with PTC and metastases to lungs, lymph nodes, and skin, was treated with five prior surgeries and two courses of RAI to a cumulative dose of 330 mCi/12.2 GBq. Due to RAI-refractory disease and rapid tumour progression, he was enrolled in a phase I clinical trial with pazopanib and trametinib; best response was stable disease. The patient’s tumour was found to have an *ETV6-NTRK3* gene fusion through FoundationOne DNA NGS and then he started larotrectinib on the phase I trial at 100 mg twice daily in September 2015. The patient had a confirmed PR after two cycles with rapid improvement in cervical lymphadenopathy and his best response was a 92.6% reduction in target lesions after 20 cycles (Fig. 4B). The patient stopped therapy in October 2020 after 65 cycles due to the progression of disease resulting from an acquired *NTRK3* solvent front mutation (p.G623R) and then switched to selitrectinib, a next-generation TRK inhibitor. The patient achieved and has maintained a durable PR on selitrectinib (currently on cycle 18 of treatment).

### Case study 3

A 13-year-old male, diagnosed at 9 years of age in 2014, was diagnosed with PTC with metastases to the lungs and lymph nodes. He had two prior surgeries, complicated by post-operative hypoparathyroidism, and two RAI treatments to a cumulative dose of 382 mCi/14.1 GBq (last treatment in 2014) with persistent but diminished tumour uptake of iodine. Despite RAI, he experienced progression of pulmonary disease (RECIST progression on the largest metastasis (shown in Fig. 4C) and new micronodular lung metastases). There was no prior systemic therapy. The patient’s tumour was found to have an *IRF2BP2-NTRK1* gene fusion through RNA NGS with Oncomine Focus assay, and he started treatment with larotrectinib in 2018 due to radiographic progression. The patient had a confirmed PR with 50% reduction in the target lesions after two cycles and then a CR in the target lesions after six cycles (Fig. 4C). The patient stopped therapy after 31 cycles due to an ongoing CR in the target lesions. His residual disease remains stable 10 months after cessation of larotrectinib.

## Discussion

Currently, the standard-of-care treatment for unselected patients with RAI-refractory advanced DTC includes the antiangiogenic multi-kinase inhibitors lenvatinib and sorafenib, which are associated with ORRs ranging from 12 to 65% (25, 26). In phase 3 clinical trials in patients with advanced DTC, lenvatinib and sorafenib demonstrated a median PFS of 18.3 and 10.8 months, respectively. Adverse events led to discontinuation of treatment in 14% of patients receiving lenvatinib and in 19% of patients receiving sorafenib. Recommended systemic treatments for patients with ATC are primarily taxanes, doxorubicin and platinum-based therapies for *BRAF* WT tumours, the dabrafenib/trametinib couplet for tumours with the *BRAF* V600E variant, and selective ALK, RET, or TRK inhibitors if an actionable fusion protein is identified (27, 28, 29).

In the current study, the highly selective TRK inhibitor larotrectinib showed durable antitumour efficacy in both adult and paediatric patients with TRK fusion-positive TC. Sustained disease control was demonstrated with a 69% 24-month PFS rate. These data exceed the outcomes reported for the non-selective oral kinase inhibitors that primarily target the vascular endothelial growth factor receptor and are widely used in RAI-refractory DTC. The antitumour efficacy of larotrectinib was demonstrated across tumour subtypes. Among patients with DTC (PTC and FTC), the ORR was 86% (95% CI: 64–97), far exceeding that in other published studies using antiangiogenic kinase inhibitors in unselected patients with DTC. While the ORR among the seven patients classified as ATC in this analysis was lower at 29% (95% CI: 4–71), response exceeds those previously reported for cytotoxic chemotherapy, immunotherapy, and lenvatinib in this population (27, 29, 30, 31). Consistent with this, recent American Thyroid Association guidelines recommend the use of a TRK inhibitor (either larotrectinib or entrectinib) in TRK fusion-positive ATC (29).

Genomic alterations are highly prevalent in ATC compared with DTC (32). Up to 95.8% of ATC cases harbour at least one genomic alteration in receptor tyrosine kinases and the PI3K/AKT and MAPK pathways. Multiple genomic alterations, especially those in *PIK3CA*, play a role in the tumourigenesis and aggressiveness of ATC (33, 34) and thus may contribute to the worse responses seen in ATC patients. However, response rates with the doublet dabrafenib/trametinib in *BRAF*-mutated ATC are very high (35), suggesting that single-agent-targeted therapy may not be sufficient in this population (36). Further studies are required to determine the mechanisms of TRK inhibitor insensitivity in TRK fusion-positive ATC patients.

In the present analysis of larotrectinib, the treatment-related AEs were mostly grades 1–2 and 2 (7%) patients experienced an AE leading to a dose reduction, 1 patient due to paraesthesia in the shoulder, and the other due to increased alanine aminotransferase. No patients experienced an AE leading to permanent discontinuation of treatment.

The multi-kinase inhibitor entrectinib, which targets TRK, ROS1, ALK, and JAK2 is another approved therapy for TRK fusion-positive solid tumours though it is limited for patients older than 12 years (37). Entrectinib demonstrated an ORR of 53.8% in 13 patients with TC of unknown subtype in a pooled analysis of three phase I/II clinical trials (ALKA-372-001, STARTRK-1, and STARTRK-2). Median DoR was 13.2 months (95% CI: 7.0–NE) (38). While this finding is lower than the 71% ORR for larotrectinib in the present analysis, it is important to note that the entrectinib data are based on a smaller patient population (*n* = 13) than for larotrectinib (*n* = 29) and thus do not allow for a head-to-head comparison. Additionally, no paediatric patients were included in the analysis of entrectinib for TC. Across the whole efficacy-evaluable study population (*n* = 121), entrectinib was associated with a median DoR of 20 months (95% CI: 13.0–38.2) and median PFS of 13.8 months (95% CI: 10.1–19.9). Among the total safety population included in the pooled analysis, entrectinib was well-tolerated with most treatment-related AEs being low grade and reversible (38). In an earlier analysis of the overall safety population (*n* = 355), the occurrence of treatment-related AEs resulting in dose reduction, dose interruption, and treatment discontinuation was 27, 25, and 4%, respectively (39).

The findings of the current analysis support routine testing for *NTRK*gene fusions in patients with advanced TC in whom systemic therapies are being considered (40, 41). All gene fusion events identified in this study were in *NTRK1*or* NTRK3*, consistent with previous data indicating that fusions in TC very rarely occur in the *NTRK2* gene, which is primarily associated with primary CNS tumours (1, 10, 11, 12, 14, 23, 42). Besides *NTRK1*and* NTRK3*, there are several other genomic alterations commonly associated with the pathogenesis of DTC, including the *BRAF^V600E^* mutation (occurring in 45% or more of adult PTCs), *RAS*gene mutations, fusions involving *RET*, *ALK, BRAF, MET, ROS1, THADA*, and the *PAX8-PPARG*fusion (occurring primarily in FTC) (43, 44, 45, 46, 47).

There are various techniques that can be used to detect *NTRK* gene fusions, such as immunohistochemistry (IHC; indirectly), fluorescence *in situ* hybridisation (FISH), and NGS. Pan-TRK IHC is rapid and inexpensive, but it is unable to distinguish between WT and chimeric TRK protein and its sensitivity with respect to TRKC fusion proteins may be low (48); thus, it may be used as a screen to identify potential cancers with a low incidence of *NTRK* gene fusions that will require subsequent confirmatory tests (48). FISH is widely available but requires multiple assays (one test per *NTRK* gene) and additional NGS to confirm the presence of an *NTRK*gene fusion; it may also be most appropriate for cancers with a high incidence of *NTRK* gene fusions (48).

Additionally, both IHC and FISH test for single biomarkers as opposed to NGS where hundreds of gene mutations and fusions can be analysed in parallel with a relatively small amount of tissue. It has been demonstrated that DNA-based sequencing techniques may not detect all gene fusions and therefore the use of RNA-based sequencing techniques may be required to support further testing to increase the chance of identifying the *NTRK* gene fusions, particularly considering the large intronic regions of *NTRK3* (48, 49). Of the 16 patients in this analysis with *NTRK3*gene fusions, 2 were *EML4-NTRK3*(both detected by RNA sequencing) and 14 were *ETV6-NTRK3*(3 detected by DNA sequencing, 7 by RNA sequencing, and 4 by both DNA and RNA sequencing). These findings support the use of RNA sequencing for detecting fusion partners with *NTRK3*(49).

In conclusion, larotrectinib is a highly active TRK inhibitor with a favourable safety profile in patients with TRK fusion-positive solid tumours. In TRK fusion-positive TC, larotrectinib results in rapid and durable disease control. Therefore, patients with advanced non-medullary TC who may require systemic therapy could be considered for testing for *NTRK* gene fusions by NGS.

## Supplementary Material

Supplementary Material

## Declaration of interest

S G W reports travel support from Bayer in the past and an active consulting agreement with Bayer (Vitrakvi expert programme). A D reports honoraria/advisory boards: Ignyta/Genentech/Roche, Loxo/Bayer/Lilly, Takeda/Ariad/Millenium, TP Therapeutics, AstraZeneca, Pfizer, Blueprint Medicines, Helsinn, Beigene, BergenBio, Hengrui Therapeutics, Exelixis, Tyra Biosciences, Verastem, MORE Health, Abbvie, 14ner/Elevation Oncology, Remedica Ltd., ArcherDX, Monopteros, Novartis, EMD Serono, Melendi, Liberum, Repare RX, Chugai, Merus, Chugai Pharmaceutical, Nuvalent, mBrace, AXIS, EPG Health, Harborside Nexus, Liberum, RV More, Ology, Amgen, TouchIME, Janssen; Associated research paid to institution: Pfizer, Exelixis, GlaxoSmithKline, Teva, Taiho, PharmaMar; Royalties: Wolters Kluwer; OTHER: Merck, Puma, Merus, Boehringer Ingelheim; CME honoraria: Medscape, OncLive, PeerVoice, Physicians Education Resources, Targeted Oncology, Research to Practice, Axis,Peerview Institute, Paradigm Medical Communications, WebMD, MJH Life Sciences, AXIS, EPG Health, JNCC/Harborside. J J L reports compensated consultant for Genentech, C4 Therapeutics, Blueprint Medicines, Nuvalent, Bayer, Novartis, and Turning Point Therapeutics; honorarium and travel support from Pfizer; institutional research funds from Roche/Genentech, Hengrui Therapeutics, Turning Point Therapeutics, Neon Therapeutics, Relay Therapeutics, Bayer, Elevation Oncology, Linnaeus Therapeutics, and Novartis; CME funding from OncLive, MedStar Health, and Northwell Health. M S B reports research support to the University of Pennsylvania School of Medicine from Bayer, Loxo Oncology, Genentech, Eisai, Blueprint Medicines, Lilly and Novartis; consultancy for Bayer, Loxo Oncology, Genentech, AstraZeneca, and Lilly, honoraria from Clinical Care Options, Medscape, OncLive and PeerView. R M reports advisory boards: Amgen, Bayer, BMS, Clovis, Janssen, Pfizer; Invited Speaker: Astellas, Ipsen, MSD; Local PI: Astellas, Bayer, BMS, Clovis, Regeneron; Coordinating PI: MSD. M A and A K have nothing to declare. J B reports advisory boards: Pfizer, Bayer, Kura, Janssen, Blueprint Medicine, Merck, Beigene, and Turning Point; Consulting: Pfizer, AstraZeneca, Lilly; Grant funding: BMS. M C reports advisory roles for Bayer, Astra-Zeneca, Pfizer, Servier, BMS, Roche. S K reports consultant/advisory board: Springworks Therapeutics, Gilead, EcoR1, Seagen, Mundibiopharma Ltd, Bayer, Oxford Biotherapeutics, Mirati, and HarbourBiomed; co-founder and equity holder for PathomlQ. spouse is a scientific advisor for Cadilla Pharmaceuticals and founder of Arxeon Inc. S L reports advisory role and travel grant support from Bayer and Immunocore. D O reports advisory/consultancy: AstraZeneca, Novartis, Genentech/Roche, Merck Serono, Bayer, Taiho, ASLAN, Halozyme, Zymeworks, Celgene, BeiGene, Basilea, Turning Point, Yuhan; research grant/funding (self): AstraZeneca, Novartis, Array, Eli Lilly, Servier, BeiGene, MSD, Handok. K P reports advisor for Loxo Oncology. D S reports consulting: Perthera, Ability Pharmaceuticals; honoraria: Foundation Medicine; and research funding: Agios, Bayer, Bristol-Myers Squibb, Celgene, Genentech, InCyte Loxo, OncoMed, Rafael. E S reports consulting: Bayer, Eisai, Merck, Eli Lilly, Roche and research: Roche, Fore Pharmaceuticals, Eli Lilly, Novartis. R N is an external employee of Bayer. J D S and N B are employees of Bayer. D S H reports Discloses research/grant funding from AbbVie, Adaptimmune, Amgen, AstraZeneca, Bayer, BMS, Daiichi-Sankyo, Eisai, Fate Therapeutics, Genentech, Genmab, Ignyta, Infinity, Kite, Kyowa, Lilly, Loxo Oncology, Merck, MedImmune, Mirati, MiRNA, Molecular Templates, Mologen, NCI-CTEP, Novartis, Pfizer, Seattle Genetics, Takeda; travel/accommodation/expenses from Loxo Oncology, MiRNA, ASCO, AACR, SITC, Genmab; consulting/advisory roles for Alpha Insights, Axiom, Adaptimmune, Baxter, Bayer, Genentech, GLG, Group H, Guidepoint Global, Infinity, Janssen, Merrimack, Medscape, Numab, Pfizer, Seattle Genetics, Takeda, and Trieza Therapeutics; ownership interests in Molecular Match (advisor), OncoResponse (founder), and Presagia Inc (advisor). M E C reports Bayer, Exelixis, Ignyta, Loxo Oncology, Blueprint, Eisai, Merck, Genentech.

## Funding

These studies were funded by Bayer http://dx.doi.org/10.13039/100004326 Healthcare and Loxo Oncology, Inc., a wholly owned subsidiary of Eli Lilly and Company http://dx.doi.org/10.13039/100004312.
