# Supplementary material for: Efficacy and safety of larotrectinib in patients with TRK fusion-positive thyroid carcinoma
Source: Eur J Endocrinol. 2022 Mar 25;186(6):631–43. doi: 10.1530/EJE-21-1259 (PMC9066591; doi:10.1530/EJE-21-1259)
Supplement: Supplementary Material [file supplementary_material.pdf]

Data supplement

Appendix 1: Study eligibility criteria

Patients were eligible for inclusion in the NAVIGATE phase II ‘basket’ trial (NCT02576431) if they had a locally advanced or metastatic solid tumour, an Eastern Cooperative Oncology Group (ECOG) performance status of 0–3, had adequate major organ function, and had received prior standard therapy appropriate for their tumour type and stage of disease, or in the opinion of the investigator, would be unlikely to tolerate or derive clinically meaningful benefit from appropriate standard-of-care therapy.

Patients with an ECOG performance status of 0–2 were eligible for inclusion in the phase I study (NCT02122913) if they had a locally advanced or metastatic solid tumour that had progressed or was nonresponsive to available therapies, were considered unfit for standard chemotherapy, or had a tumour for which no standard or available curative therapy exists.

Children with adequate haematologic, hepatic, and renal function were eligible for inclusion in the SCOUT phase I/II study (NCT02637687) if they had a locally advanced or metastatic solid tumour or primary central nervous system tumour that had relapsed, progressed, or was nonresponsive to available therapies and for which no standard or available systemic curative therapy exists.

Supplementary Table 1: Patient/tumour characteristics and prior therapies

**Patient**	**Age of patient at enrolment**	**Subtype**	**Cancer histology**	**Fusion**	**Fusion partner**	**Most recent prior systemic regimen**	**RAI**	**External RT**
1	50	Differentiated	Papillary	*NTRK3*	*ETV6*	N/A	Y	Y
2	68	Differentiated	Papillary	*NTRK3*	*EML4*	Cabozantinib	Y	Y
3<br>Case study 2	33	Differentiated	Papillary	*NTRK3*	*ETV6*	Pazopanib + trametinib	Y	N
4	80	Differentiated	Papillary	*NTRK3*	*ETV6*	N/A	Y	Y
5*‡	68	Differentiated	Papillary	*NTRK3*	*ETV6*	Pembrolizumab	Y	Y
6	29	Differentiated	Papillary	*NTRK1*	*DIAPH1*	N/A	Y	N
7‡<br>Case Study 1	66	Differentiated (PDTC)	Papillary	*NTRK3*	*ETV6*	Ipilimumab	Y	Y
8	27	Differentiated	Papillary	*NTRK1*	*TPM3*	Lenvatinib	Y	N

9[†]<br><br>Case study 3	13	Differentiated	Papillary	*NTRK1*	*IRF2BP2*	Unknown	Y	N
10	72	Differentiated	Papillary	*NTRK3*	*ETV6*	Lenvatinib	Y	Y
11	52	Differentiated	Papillary	*NTRK3*	*ETV6*	Lenvatinib	Y	N
12*	47	Differentiated	Papillary	*NTRK3*	*ETV6*	N/A	Y	Y
13	65	Differentiated	Papillary	*NTRK1*	*PPL*	Trametinib	Y	N
14	74	Differentiated	Papillary	*NTRK3*	*ETV6*	N/A	Y	Y
15	63	Differentiated	Papillary	*NTRK1*	*TPR*	N/A	Y	N
16	60	Differentiated	Papillary	*NTRK1*	*TPM3*	N/A	Y	Y
17	75	Differentiated	Papillary	*NTRK3*	*ETV6*	Sorafenib	Y	N
18*	55	Differentiated	Papillary	*NTRK1*	*TPR*	Lenvatinib	Y	Y
19[†]	6	Differentiated	Papillary	*NTRK1*	*TPR*	N/A	Y	N
20	18.5	Differentiated	Papillary	*NTRK1*	*TPR*	N/A	Y	N

21*	63	Differentiated	Follicular	*NTRK3*	*ETV6*	Sunitinib	N	Y
22	56	Differentiated	Follicular	*NTRK1*	*TPM3*	Lenvatinib	Y	Y
23	69	Undifferentiated (PDTC)	Anaplastic	*NTRK3*	*EML4*	N/A	Y	N
24	57	Undifferentiated	Anaplastic	*NTRK1*	*IRF2BP2*	Cisplatin	N	Y
25	64	Undifferentiated (PDTC)	Anaplastic	*NTRK1*	*NFASC*	N/A	Y	Y
26	69	Undifferentiated	Anaplastic	*NTRK3*	*ETV6*	Paclitaxel	N	Y
27	49	Undifferentiated	Anaplastic	*NTRK1*	*TPM3*	Doxorubicin	N	Y
28	77	Undifferentiated	Anaplastic	*NTRK3*	*ETV6*	N/A	N	Y
29	52	Undifferentiated	Anaplastic	*NTRK3*	*ETV6*	N/A	N	N

*Patients with CNS metastases at baseline. [†]Paediatric patients (<18 years of age). [‡]Patients received immunotherapy as prior systemic therapies. CNS, central nervous system; PDTC, poorly differentiated thyroid carcinoma; RAI, radioactive iodine; RT, radiotherapy.
